# A Cyanide-Free UHPLC-MS/MS Workflow for the Analysis of Major Cobalamin Vitamers in Foods

**DOI:** 10.3390/foods15091506

**Published:** 2026-04-26

**Authors:** Fengen Wang, Li Cao, Min Ding, Ruiju Li, Chao Zhang, Baorui Li, Zhaowei Yang, Kaizhen Liu, Jiamei Xin, Xia Li, Tongcheng Xu, Ligang Deng

**Affiliations:** 1 Institute of Agricultural Products Quality Safety and Standards, Shandong Academy of Agricultural Sciences, Jinan 250100, China; wfe8520382@163.com (F.W.); cl1809442973@163.com (L.C.); dingminzhen@163.com (M.D.); liruiju-98@163.com (R.L.); ytzc1212@163.com (C.Z.); lisa-fsd@163.com (X.L.); 2 College of Animal Science and Technology, Henan Agricultural University, Zhengzhou 450046, China; 18728187930@163.com; 3 Institute of Food and Nutrition Science and Technology, Shandong Academy of Agricultural Sciences, Jinan 250100, China; lbr_simmer@126.com (B.L.); xjm0312@163.com (J.X.); xtc@live.com (T.X.); 4 Qinhuangdao Agriculture Product Quality Safety Inspection and Monitoring Center, Qinhuangdao Municipal Bureau of Agriculture and Rural Affairs, Qinhuangdao 066099, China; yangzhaowei1984@163.com

**Keywords:** cobalamin vitamers, UHPLC-MS/MS, cyanide-free analysis, light stability, tandem cleanup, food matrices

## Abstract

Accurate determination of cobalamin vitamers in foods remains analytically challenging because conventional cyanidation-based methods convert native cobalamins into cyanocobalamin (CNCbl) and may distort their original distribution. In this study, a cyanide-free UHPLC-MS/MS workflow was developed for the analysis of major cobalamin vitamers in foods, with particular emphasis on preserving native forms during sample preparation. Light, temperature, and cleanup procedures were systematically evaluated. Methylcobalamin (MeCbl) and adenosylcobalamin (AdoCbl) showed pronounced light sensitivity, whereas red-light handling better preserved vitamer integrity during pre-analytical operations. A tandem cleanup procedure combining immunoaffinity and Oasis HLB solid-phase extraction improved extract cleanliness in complex food matrices. The workflow showed good chromatographic separation and excellent linearity (R^2^ > 0.999). The validated limits of detection were 0.5 μg/kg for CNCbl, 1.0 μg/kg for AdoCbl, and 0.75 μg/kg for MeCbl. Application to food samples showed no detectable target cobalamins in the tested plant-derived foods, whereas animal liver and oyster samples showed comparatively high levels of the target cobalamin vitamers, with AdoCbl predominating in liver. The proposed workflow may serve as a practical cyanide-free option for exploratory or comparative native-vitamer analysis of CNCbl, AdoCbl, and MeCbl in foods within the current validation scope, particularly when full sets of matched isotope-labeled standards are not readily available.

## 1. Introduction

**Figure 1:**
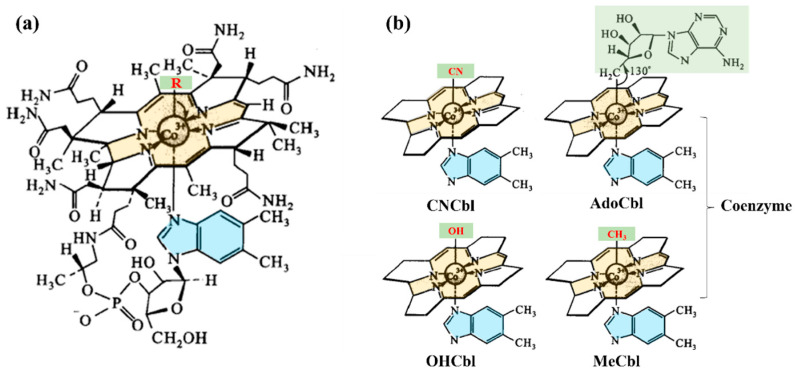
(**a**) Chemical structure of cobalamin; (**b**) Simplified structures of four major cobalamin vitamers: CNCbl, AdoCbl, MeCbl and OHCbl.

Vitamin B12 (cobalamin) is an essential water-soluble vitamin and the only known cobalt-containing cofactor in human metabolism [1,2]. Structurally, as shown in Figure 1a, cobalamins consist of a corrin ring coordinated to a central cobalt atom, with variable upper axial ligands and, in canonical cobalamins, 5,6-dimethylbenzimidazole as the lower axial ligand. The vitamin occurs in several forms depending on the upper axial ligand, as shown in Figure 1b, primarily cyanocobalamin (CNCbl), methylcobalamin (MeCbl), 5′-deoxyadenosylcobalamin (AdoCbl), and hydroxocobalamin (OHCbl) [1,2].

Although CNCbl is not itself a biologically active cofactor, it is the most stable cobalamin form and is therefore widely used in food fortification and pharmaceutical supplementation [2,3,4]. In vivo, CNCbl is enzymatically converted into the bioactive cofactor forms MeCbl and AdoCbl through stepwise reduction in the cobalt center [5,6]. MeCbl functions as the cofactor of methionine synthase, supporting the remethylation of homocysteine to methionine and thereby contributing to one-carbon metabolism and DNA methylation [7,8], whereas AdoCbl serves as the cofactor of methylmalonyl-CoA mutase, catalyzing the conversion of methylmalonyl-CoA to succinyl-CoA in mitochondrial propionate metabolism [5,9]. Through these roles, vitamin B12 is essential for normal metabolic, hematological, and neurological function [4]. Accordingly, vitamin B12 deficiency has been associated with hyperhomocysteinemia, megaloblastic anemia, and neurological dysfunction, and is also indirectly linked to impaired myelin integrity through disturbances in methylmalonyl-CoA metabolism and related metabolic processes [2,10,11,12,13,14,15,16,17].

Although certain gut microorganisms can synthesize cobalamin, dietary intake remains the primary source of biologically relevant vitamin B12 in humans [7,18,19]. Animal-derived foods are the major natural dietary sources, but their cobalamin content varies considerably by matrix: muscle meats generally contain modest levels, organ meats such as liver and kidney contain much higher concentrations, milk contains measurable but relatively low amounts, and aquatic products—especially shellfish—are often particularly rich sources [20,21,22,23,24,25,26,27,28]. However, reported values can vary markedly across species and studies, and in some cases exceed plausible biological variation, indicating that structural confirmation is necessary, especially in marine foods where corrinoid analogs may contribute to analytical overestimation [26,28,29,30]. By contrast, most staple plant foods contain negligible or non-detectable amounts of bioactive vitamin B12 because higher plants lack the biosynthetic pathway for cobalamin production, while reported cobalamin activity in certain seaweeds or fermented plant products remains highly variable and requires cautious interpretation because inactive corrinoid analogues may be present [3,19,31,32,33]. As a result, plant-based diets inherently provide limited vitamin B12, increasing deficiency risk in populations such as vegetarians and elderly individuals with impaired absorption capacity, and thereby highlighting the nutritional importance of cobalamin fortification and supplementation [3,31,34]. Among available vitamers, CNCbl is most commonly used in fortified foods because of its superior processing and storage stability, whereas in natural food matrices—particularly animal-derived products—vitamin B12 mainly occurs as the bioactive forms MeCbl and AdoCbl [5,11]. Accurate vitamer-resolved determination is therefore important for evaluating the nutritional value of foods, characterizing fortified products, and assessing structural changes associated with processing.

Traditionally, cobalamin quantification relied on microbiological assays, which are activity-based, labor-intensive, and unable to distinguish native cobalamin vitamers from inactive corrinoid analogs such as pseudovitamin B12 [2,35,36]. Liquid chromatography subsequently became the preferred analytical platform and was adopted in standardized methods, typically involving enzymatic release of protein-bound cobalamin, buffer extraction, solid-phase cleanup, and chromatographic determination [2,37,38]. However, because MeCbl and AdoCbl are highly light-sensitive, cyanide reagents are commonly used to convert native cobalamins into the more stable CNCbl before IAC enrichment and total-content measurement, which improves stability but obscures native vitamer distribution [2,4]. The introduction of LC–MS and LC–MS/MS shifted cobalamin analysis from activity-based or cyanide-converted total measurement toward structurally defined, vitamer-resolved determination, enabling the discrimination of individual cobalamins and reducing interference from pseudo-B12 and complex matrices [22,30,39,40,41]. Subsequent studies further improved selectivity and sensitivity, and stable isotope dilution (SIDA) LC–MS/MS markedly enhanced trueness by correcting for recovery loss and ion suppression [1,42,43,44]. Nevertheless, SIDA remains constrained by the limited availability, high cost, and synthetic complexity of labeled cobalamin standards, and it cannot retrospectively correct interconversion or degradation occurring before isotope equilibration [45,46,47]. As a result, many laboratories still rely on single-standard or non-vitamer-matched calibration, making quantitative results highly sensitive to extraction control, structural stability management, and purification selectivity rather than detector sensitivity alone [22,39,47].

Against this background, the key methodological challenge is not merely the detection of cobalamin, but the structurally resolved determination of native vitamers in complex food matrices while minimizing cyanidation-induced information loss, pre-analytical instability, purification bias, and vitamer-dependent recovery differences. In particular, routine analysis still faces three practical constraints: preservation of light-sensitive native forms during sample preparation, achievement of acceptable cleanup and recovery across different vitamers, and maintenance of quantitative reliability when fully matched isotope-labeled standards are not available [4,22,43,45,46,47].

In response to these issues, the present study describes a cyanide-free UHPLC-MS/MS workflow for the analysis of major cobalamin vitamers in foods, with particular emphasis on the nutritionally active MeCbl and AdoCbl and the commonly fortified form CNCbl. By eliminating cyanidation-induced artificial conversion, systematically evaluating light- and temperature-dependent degradation, and introducing a tandem cleanup strategy, the present study was designed to explore a cyanide-free analytical route for native-vitamer analysis in complex food matrices under practical laboratory constraints, while attempting to preserve native vitamer information during sample preparation.

## 2. Materials and Methods

### 2.1. Chemicals and Reagents

Acetonitrile (HPLC grade), methanol (HPLC grade), formic acid (HPLC grade) and ammonium formate (≥99.0%) were obtained from Thermo Fisher Scientific Inc. (Waltham, MA, USA). Acetic acid (analytical grade) and sodium acetate anhydrous (≥99.0%) were purchased from Sinopharm Chemical Reagent Co., Ltd. (Shanghai, China). Pepsin (USP grade, activity 1:3000), α-amylase (≥5 U/mg) and Soy protein powder (≥90.0%) for spike recovery tests were purchased from Yuanye Bio-Technology Co., Ltd. (Shanghai, China). Ultrapure water was prepared using a Milli-Q purification system (Merck Millipore, Darmstadt, Germany).

The reference standards used in this study were obtained from Yuanye Bio-Technology Co., Ltd. (Shanghai, China), including CNCbl (≥99%), MeCbl (≥96%), OHCbl (≥97%), AdoCbl (≥98%). The internal standard vitamin B12−^13^C_7_ (100 µg/L, ^13^C_7_-CNCbl) was obtained from Tianjin Boner-Agela Technologies Co., Ltd. (Tianjin, China).

### 2.2. Samples Collection

To validate the applicability of the established method, representative samples were collected and analyzed. Plant-derived food samples, including 8 wheat flour, 3 maize flour, 3 millet, and 3 peanut samples, were collected from local markets in Shandong province, China in 2025. 7 wild jujube tea samples were obtained from markets in Shandong, Shaanxi, and Hebei provinces in 2024. Animal-derived food samples, including 3 chicken liver, 5 pork liver, 5 lamb liver, 3 oyster, and 3 sea cucumber samples, were all procured from local markets in Shandong province in 2025. All samples were thoroughly homogenized after collection (powdered samples were mixed evenly, and animal-derived tissues were minced or blended to obtain uniform material) and then immediately stored at −20 °C until analysis.

### 2.3. Stability Tests of Vitamers

A photostability study was conducted to assess the vitamers (CNCbl, MeCbl, AdoCbl, and OHCbl). Each vitamer’s solid powder was weighed in an amber volumetric flask under light-protected conditions and dissolved in water to prepare individual standard solutions. After transferring to clear glass beakers for light exposure, samples were collected at 1, 10, 60, and 120 min into amber HPLC vials. The light sources include sunlight, purple LED spotlights (380–390 nm, 20 W, Jiadeng, Xuzhou, China), yellow-green LED spotlights (560–580 nm, 20 W, Jiadeng, Xuzhou, China) and red LED spotlights (670–680 nm, 20 W, Jiadeng, Xuzhou, China). Quantitative analysis was performed using UHPLC-MS/MS to determine concentration changes. All measurements were performed in triplicate.

Thermal stability testing was also conducted. Each individual standard solution was aliquoted and incubated in water baths at 40 °C, 60 °C, 80 °C, and 100 °C, separately. Samples were collected at 1, 10, and 60 min into amber HPLC vials for analysis. Quantitative analysis was performed using UHPLC-MS/MS to determine concentration changes. All measurements were performed in triplicate. The thermal stability assay was included to assess short-term vitamer stability over the time scale relevant to conventional high-temperature treatment steps used in some cobalamin methods and to determine whether temperature represented a practical risk factor during method development.

### 2.4. Retention Performance Tests of Solid-Phase Extraction Columns

IACs for cobalamin were purchased from Pribolab PTE. Ltd. (Qingdao, China) and stored at 2–8 °C. Before use, the columns were equilibrated to room temperature, and the storage buffer was discarded. To evaluate purification behavior, 3 mL of mixed standard solution (100 µg/L for each vitamer) was loaded onto the column, followed by rotation and shaking for 15 min to ensure sufficient interaction between the target analytes and the antibodies. The loading effluent was then collected sequentially into three 1 mL amber HPLC vials. Subsequently, 3 mL of water was used for washing, and the wash solution was likewise collected in three 1 mL fractions. After complete drainage, the bound analytes were eluted with 3 mL of methanol, which was also collected in three separate 1 mL fractions.

Other solid-phase extraction (SPE) columns, including Oasis HLB (3 mL, 60 mg) and Oasis PRiME HLB (3 mL, 60 mg), were obtained from Waters Corporation (Milford, MA, USA). Captiva EMR (3 mL, 300 mg) was purchased from Agilent Technologies (Santa Clara, CA, USA), while Bond Elut HLB (3 mL, 60 mg) was acquired from Agilent Technologies (Beijing, China). Prior to extraction, the SPE columns were activated with 5 mL of water. To evaluate purification behavior, 3 mL of mixed standard solution (100 µg/L for each vitamer) was then loaded, and the flow-through was collected sequentially into three 1 mL amber HPLC vials. This was followed by a 3 mL water wash, which was likewise collected in three 1 mL fractions. After complete drainage, the analytes were eluted with 3 mL of methanol, collected in three separate 1 mL fractions.

Each fraction was analyzed individually by UHPLC-MS/MS to characterize the stepwise distribution of individual vitamers during cleanup, including retention, breakthrough, and elution behavior. All measurements were performed in triplicate.

### 2.5. Sample Preparation

The frozen samples were completely thawed at room temperature prior to analysis. Based on AOAC Official Method 2014.02 [37], we optimized the sample preparation procedure for the determination of major cobalamin vitamers while minimizing structural interconversion during sample preparation. Notably, most operations were performed under light-protected conditions, including weighing and pipetting in a darkroom, using amber-colored centrifuge tubes and HPLC vials, and wrapping SPE columns with aluminum foil throughout the process. During nitrogen evaporation, the sample was briefly inspected under red LED light to monitor whether the extract had nearly reached dryness.

For complete extraction of vitamers, 5 g of homogenized sample was spiked with 100 µL of ^13^C_7_-CNCbl internal standard solution (100 µg/L), followed by the addition of 15 mL sodium acetate buffer (0.25 mol/L, pH 4~5), 100 µL pepsin aqueous solution (400 mg/mL), and 100 µL α-amylase aqueous solution (100 mg/mL). The mixture was incubated at 37 °C with continuous shaking for 30 min of enzymatic digestion. After enzymatic treatment, the samples were centrifuged at 10,000× *g* for 5 min at 4 °C, and the supernatant was collected for subsequent SPE purification.

A tandem purification strategy employing the IACs coupled with the Oasis HLB column was implemented to effectively remove matrix interferences while retaining target compounds. The refrigerated IACs were equilibrated to room temperature, and the storage buffer was discarded. Subsequently, 3 mL of supernatant was loaded onto each IAC, followed by 15 min of rotational mixing in a sealed system. For tandem purification, the IAC column was connected in series with an Oasis HLB column, and the loading solution was slowly eluted from the IAC column directly onto the Oasis HLB column. Sequential washing was then performed with 3 mL of water through both columns, with all wash fractions being discarded before complete drainage of both columns. For final elution, the Oasis HLB column and IAC column were reconnected in reverse orientation, and target compounds were eluted with 3 mL of methanol delivered in three sequential aliquots. The collected eluate was blown to near-dryness under nitrogen, reconstituted with 0.5 mL of 5 mM ammonium formate aqueous solution, and filtered through a 0.45-μm aqueous membrane prior to UHPLC-MS/MS analysis.

Pepsin was retained in the extraction procedure as a practical digestion aid to facilitate the release of protein-bound cobalamin, particularly from protein-rich food matrices. We do not suggest that pepsin is universally optimal for all matrices; rather, under the present experimental conditions, it provided acceptable analyte release when combined with α-amylase-assisted extraction.

### 2.6. UHPLC-MS/MS Detection

The vitamers were determined using an Agilent 1290 Infinity II UHPLC system equipped with a 6475 MS detector (Agilent Technologies, Santa Clara, CA, USA) and an Agilent AQ-C18 column (2.1 × 100 mm, 2.7 μm). The proposed method employed UHPLC gradient separation and MS/MS multiple-reaction monitoring, and the detailed parameters are shown in Table 1.

### 2.7. Method Validation

Sensitivity, linearity, accuracy, and precision were evaluated for method validation [48]. Sensitivity was determined by signal-to-noise ratios of 3 and 10, corresponding to the instrument detection limit (IDL). Subsequently, the limit of detection (LOD) was determined by accounting for sample pretreatment dilution factors and matrix effects, while the limit of quantification (LOQ) was established accordingly. For each vitamer, calibration curves were constructed using 7 concentration levels (5, 20, 50, 200, 500, 2000, 5000 µg/L) to establish the linear range and regression equation. The linearity of fit was evaluated by the coefficient of determination (R^2^).

To evaluate accuracy and precision, blank soy protein powder was used as a practical surrogate matrix for fortified QC preparation and was spiked with CNCbl, AdoCbl, MeCbl, and OHCbl at three concentrations (2, 10, and 50 μg/100 g), with three replicate analyses performed at each level. The spike-recovery levels were selected to broadly cover the approximate concentration range reported in representative food matrices, from ordinary meat products to liver [23], and thus to reflect low, intermediate, and relatively high practical concentration levels in food analysis. The fortified materials were subjected to the same extraction, tandem cleanup, and UHPLC-MS/MS workflow as the real samples. Recoveries and coefficients of variation (CV) were calculated to assess method applicability under the current validation scope.

### 2.8. Quantification and Quality Control

For method validation and sample analysis, ^13^C_7_-CNCbl was used as the only available internal standard to compensate, at least in part, for analyte loss during extraction and cleanup. However, because only one internal standard was available, full vitamer-specific correction across all analytes could not be established. In particular, ^13^C_7_-CNCbl does not fully mimic the extraction, cleanup, and ionization behavior of AdoCbl and MeCbl. Therefore, the correction provided by ^13^C_7_-CNCbl should be regarded as partial procedural compensation rather than analyte-matched quantitative normalization across all vitamers.

In principle, matrix effects can be evaluated by comparing solvent-based and matrix-matched calibration curves. However, true blank animal-derived matrices for native vitamer analysis were difficult to obtain, particularly for liver and aquatic products, because endogenous background levels were often substantial. Therefore, a full slope-based matrix-effect evaluation was not established in the present study. Instead, blank soy protein powder was used as a practical surrogate QC matrix and was fortified with the four cobalamins at 2, 10, and 50 μg/100 g. The QC samples used for recovery-based correction were the same fortified soy protein powder materials as those used in the spike recovery experiments. These QC samples were analyzed concurrently with real samples, and sample concentrations were corrected according to the recovery obtained from the QC level closest to the analyte concentration.

### 2.9. Statistical Analysis

All experiments were performed in triplicate unless otherwise stated, and the results are presented as mean ± standard deviation (SD). Data processing and organization were conducted using Excel 2019. Because the study focused on method development and validation, analytical performance was evaluated descriptively on the basis of concentration changes, recovery values, and coefficients of variation under the defined experimental conditions. Dynamic heat map and principal component analysis (PCA) were performed using the OmicShare tools, a free online platform for data analysis (https://www.omicshare.com/tools/, accessed on 16 April 2026). PCA was conducted based on the contents of CNCbl, AdoCbl, and MeCbl in the 19 animal-derived samples. The loading vectors were derived from the component matrix generated by SPSS Statistics 26.0 (IBM, Armonk, NY, USA), and the PCA biplot was constructed accordingly.

## 3. Results and Discussion

### 3.1. The Selectivity of the UHPLC-MS/MS Method

**Figure 2:**
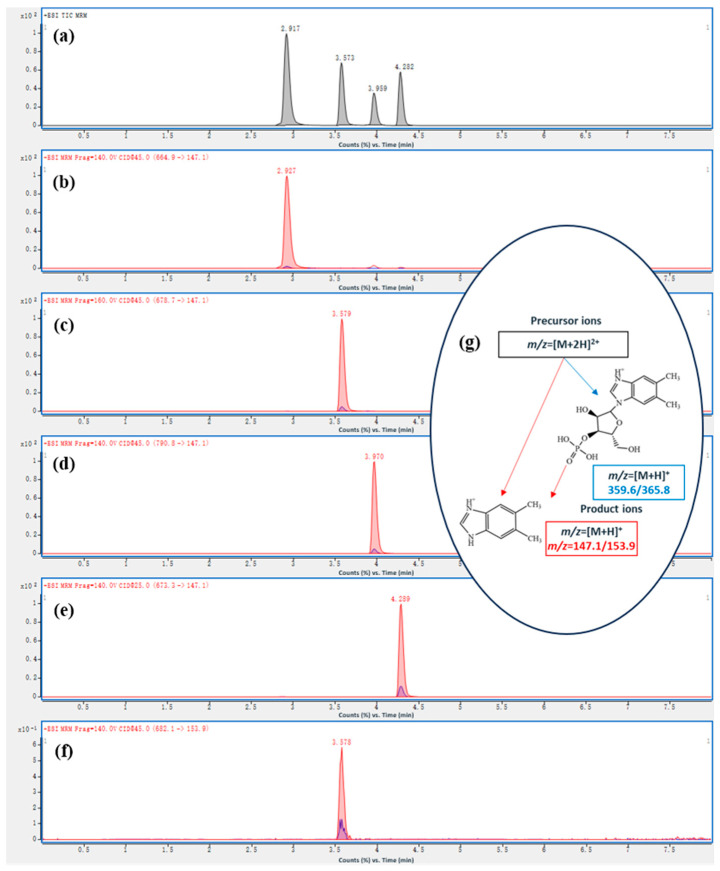
(**a**) Total ion chromatograms (TIC) of the mixed standard solution of four cobalamins (3 mg/L); (**b**) Selected ion chromatograms (SIC) of OHCbl; (**c**) SIC of CNCbl; (**d**) SIC of AdoCbl; (**e**) SIC of MeCbl; (**f**) SIC of ^13^C_7_-CNCbl (3 µg/L); (**g**) The mechanism of the formation of characteristic ion pairs.

As shown in Figure 2a, baseline separation with excellent peak symmetry was achieved for the targeted cobalamin vitamers (OHCbl, CNCbl, AdoCbl, and MeCbl) on the liquid chromatography column. Their respective retention times were 2.96 min for OHCbl, 3.59 min for CNCbl, 3.96 min for AdoCbl, and 4.29 min for MeCbl. Compared with the previously reported method of Fan et al. [43], the present method showed improved chromatographic separation of the target vitamers.

For each vitamer, characteristic ion pairs were carefully selected and optimized by adjusting declustering potential (DP) and collision energy (CE) to enhance signal intensity. As shown in Figure 2b–e, the doubly charged precursor ions ([M+2H]^2+^) were selected according to their signal abundance and stability. For CNCbl (*m*/*z* 678.7), AdoCbl (*m*/*z* 790.8), and MeCbl (*m*/*z* 673.3), the intact [M+2H]^2+^ ions were used as precursor ions. For OHCbl, the theoretical [M+2H]^2+^ ion is *m*/*z* 673.8. However, OHCbl is prone to in-source fragmentation, with loss of the upper hydroxyl ligand resulting in dehydration (−18 Da), generating a fragment ion at *m*/*z* 664.9. Therefore, although OHCbl was included in chromatographic separation and signal monitoring, its quantitative results should be interpreted with greater caution than those of CNCbl, AdoCbl, and MeCbl under the current method configuration.

Notably, AdoCbl demonstrated significantly stronger signal response for [M+2H]^2+^ (*m*/*z* 790.8) compared to the previously reported [M+3H]^3+^ (*m*/*z* 527) [43]. As illustrated in Figure 2g, two product ions (*m*/*z* 147.1 and 359.2) were consistently selected across all analytes, corresponding to dimethylbenzimidazole and ribose-phosphate-dimethylbenzimidazole fragments generated during MS ionization, which aligns with AOAC standards and published literature [37,43]. As shown in Figure 2f, for the internal standard CNCbl-^13^C_7_, the quantitative and qualitative ion pairs were *m*/*z* 682.1 → 153.9 and *m*/*z* 682.1 → 365.8, respectively. As a result, the established UHPLC-MS/MS workflow provided good selectivity for the major cobalamin vitamers.

### 3.2. The Stability of the Vitamers

Since the publication of a report on CNCbl photolysis under various radiation types [49], debates regarding cobalamin photostability have persisted. While consensus exists that the coenzyme-active forms AdoCbl and MeCbl undergo more rapid photodegradation [4,50], the relative stability of OHCbl versus CNCbl remains contentious [50,51].

**Figure 3:**
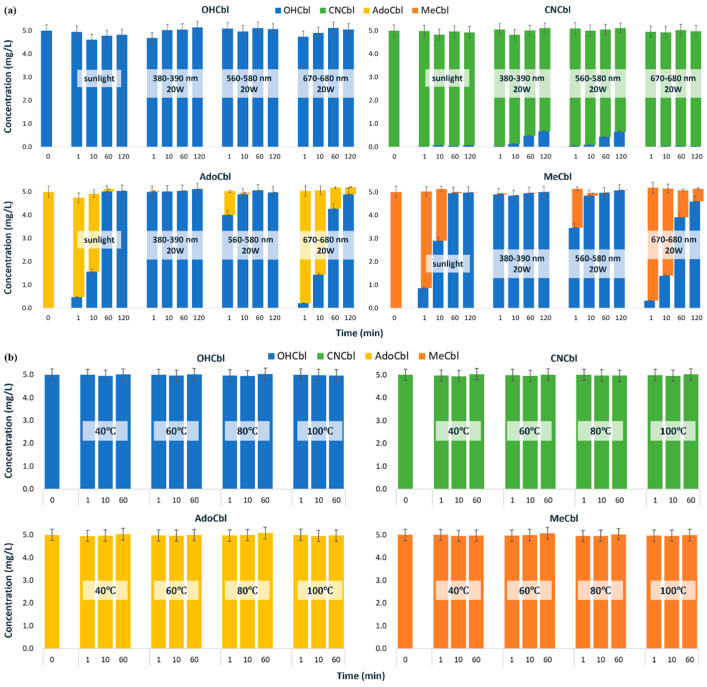
Stability tests of the vitamers (*n* = 3). (**a**) Photostability test: OHCbl and CNCbl maintained their chemical structures under light exposure, especially under sunlight and red LED spotlight (670–680 nm), while AdoCbl and MeCbl were extensively converted to OHCbl, especially under yellow-green LED spotlight (560–580 nm) and purple LED spotlight (380–390 nm). (**b**) Thermal stability test: no observable degradation or interconversion was detected for OHCbl, CNCbl, AdoCbl, or MeCbl during 60 min at 40–100 °C under the tested conditions.

Our photostability tests (Figure 3a) confirmed distinct behaviors among the vitamers: OHCbl and CNCbl demonstrated superior photostability, whereas AdoCbl and MeCbl exhibited marked conversion to OHCbl. Accounting for concentration changes from solvent evaporation and analytical variability, OHCbl showed negligible degradation under either sunlight or various types of LED lighting, with its breakdown pathway involving irreversible transformation to unidentified porphyrin oxidation products [52,53]. CNCbl similarly displayed minimal degradation under either sunlight or red LED lighting (670–680 nm, 20 W), with trace OHCbl generation attributed to hydroxyl group substitution of the cyano moiety in neutral solutions [52,53]. However, upon exposure to purple (380–390 nm, 20 W) and yellow-green LED lights (560–580 nm, 20 W), CNCbl was observed to convert stepwise to OHCbl, with conversion rates of approximately 1.8–2.6% and 12.8–13.6% after 10 and 120 min of irradiation, respectively. In comparison, the photochemical conversion of AdoCbl and MeCbl to OHCbl proceeded more rapidly and to a greater extent. This stems from their photolytic reduction to cob (II)alamin (vitamin B12r), which rapidly oxidizes to OHCbl at neutral pH [54,55,56]. Quantitative analysis revealed that AdoCbl and MeCbl were rapidly converted to OHCbl under both purple and yellow-green LED lighting, with nearly complete conversion occurring within 1 min under purple lighting or 10 min under yellow-green lighting. Under sunlight exposure, the conversion rate decreased; however, nearly complete conversion of both AdoCbl and MeCbl to OHCbl was still achieved within approximately 60 min. In contrast, the highest photostability was observed under red light. The conversion ratios to OHCbl for AdoCbl at 1, 10, 60, and 120 min were 4.2%, 28.6%, 85.6%, and 98%, respectively, while those for MeCbl under the same conditions were 6.4%, 28.0%, 78.2%, and 91.8%. These results indicate that the photochemical behaviors of AdoCbl and MeCbl are similar, which contrasts with the previously reported greater photosensitivity of MeCbl (e.g., a 3-fold higher susceptibility to UVA radiation compared to AdoCbl) [50]. Discrepancies in reported degradation rates arise from methodological variations, particularly in solution pH and light exposure conditions (wavelength range and irradiance), which critically influence degradation kinetics [4]. Given the marked photosensitivity of cobalamins, especially AdoCbl and MeCbl, strict light-protective measures must be implemented during sample preparation. This includes all post-hydrolysis extraction and purification steps. When brief visual inspection is necessary (e.g., to confirm the endpoint of nitrogen blowing), it should be performed rapidly under red light to ensure the chemical stability of the target compounds.

Existing studies indicate that temperature can affect the stability of cobalamins in aqueous solution [57]. CNCbl shows limited degradation at room temperature, but more pronounced loss at elevated temperature over prolonged incubation, and acidic conditions can further accelerate thermal degradation [57,58]. In light-protected systems, the thermal stability of MeCbl and AdoCbl has been reported to be broadly similar to that of CNCbl [35,59], whereas the relative thermal stability of OHCbl remains less consistent across studies [51,60,61]. In the present study, however, no observable degradation or interconversion was detected for the major vitamers during 60 min at 40–100 °C (Figure 3b). These results suggest that, within the short time scale relevant to the present workflow, temperature was less critical than light exposure for preserving native vitamer integrity.

### 3.3. Purification Strategy and Approach

The complex composition of food matrices, rich in carbohydrates, lipids, proteins, and pigments, poses significant challenges for accurate quantification, necessitating effective removal of potential contaminants and enhancement of target analyte purity during sample cleanup [36]. Previous studies typically employed either C18 columns [22,40] or vitamin B12-specific IACs [21,62,63] for single purification following extraction and cyanidation, primarily targeting CNCbl quantification. However, when the analytical focus shifted to simultaneous determination of multiple vitamers, researchers recognized the critical need to evaluate purification efficacy across diverse matrices for each vitamer [43,44]. Researchers observed asynchronous behavior among vitamers during purification, resulting in differential column retention efficiencies, which prompted the development of combined SPE purification strategies, such as the tandem configuration of IAC columns with Bond Elut Plexa columns [1].

**Figure 4:**
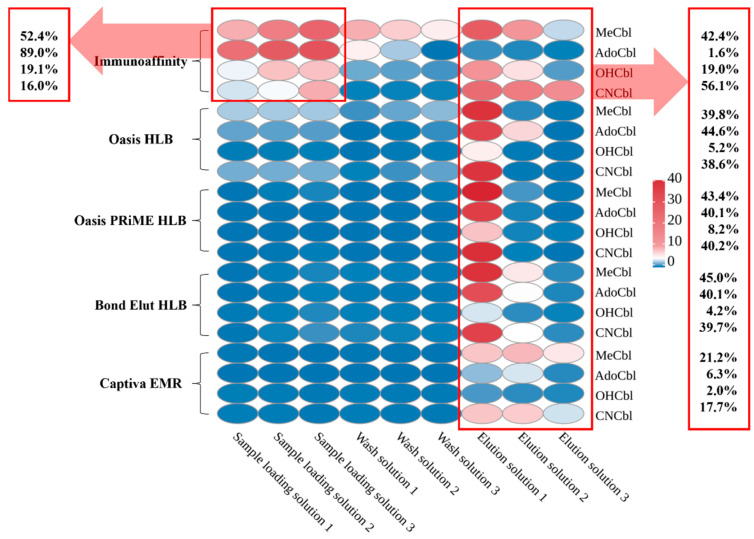
Comparison and selection of purification columns: The IAC demonstrated significant analyte loss during the loading phase, particularly for AdoCbl and MeCbl, but exhibited good specificity and high elution recovery for CNCbl. Although Oasis HLB, Oasis PRiME HLB, Bond Elut HLB, and Captiva EMR columns showed minimal losses during loading and washing steps, they yielded relatively low recoveries during elution, especially for OHCbl.

In this study, a vitamin B12 immunoaffinity column (IAC) and several SPE columns commonly used for animal-derived food cleanup were compared for vitamer retention behavior. To quantify purification performance, the loading effluent, washing fractions, and elution fractions were collected on a per-mL basis and analyzed individually. Thus, Figure 4 represents the stepwise distribution of each vitamer during cleanup and provides a purification-stage mass-distribution profile of retention, breakthrough, and elution behavior. The heat map in Figure 4 was constructed from the mean values of three replicate measurements and was intended to visualize the fraction-distribution pattern of each vitamer during purification, rather than to support inferential statistical comparison.

As shown in Figure 4, the IAC exhibited a distinct vitamer-dependent retention pattern. During sample loading, substantial losses of AdoCbl and MeCbl were observed, reaching 89.0% and 52.4%, respectively, which resulted in only 1.6% and 42.4% being finally retained in the elution fractions. By contrast, CNCbl showed minimal loss during the loading and washing steps and therefore displayed the highest retention in the elution fractions, reaching 56.1%. These results indicate that the IAC column showed clear selectivity toward CNCbl but insufficient retention for the native vitamers, particularly AdoCbl.

The other SPE columns showed broadly similar retention behavior, except for Captiva EMR, which exhibited generally poorer overall retention. For Oasis HLB, Oasis PRiME HLB, and Bond Elut HLB, losses of CNCbl, AdoCbl, and MeCbl during loading and washing were relatively limited, whereas the retained proportion in the elution fractions was typically around 38.6–45.0%. Notably, AdoCbl showed substantially better retention on these SPE columns than on the IAC, indicating that these sorbents could recover the fraction insufficiently retained by IAC during loading. This was an important reason for selecting Oasis HLB as the complementary purification column in the tandem strategy.

OHCbl showed unfavorable retention behavior on both IAC and SPE columns. Its poor recovery was not mainly reflected as a simple breakthrough during loading or washing, but rather as failure to be effectively recovered in the final elution fractions, which may be attributed to strong adsorption to the sorbent and irreversible degradation during purification [52,53].

Based on these results, a tandem purification strategy combining IAC with Oasis HLB was adopted. The IAC showed relatively higher recovery for CNCbl, whereas Oasis HLB provided substantially better recovery for AdoCbl and comparable recovery for MeCbl. Therefore, Oasis HLB was used to recover vitamers insufficiently retained by the IAC column, especially AdoCbl lost during loading. Under the tandem cleanup procedure described in Section 2.5, the overall recoveries before internal standard correction were approximately 49.7% for AdoCbl, 57.3% for MeCbl, and 77.4% for CNCbl, whereas OHCbl remained below 20% and showed poor stability. These overall recoveries were generally consistent with the fraction-distribution patterns observed in Figure 4, indicating that the stepwise purification assessment realistically reflected cumulative vitamer-dependent loss during the actual workflow.

This tandem IAC-HLB workflow improved cleanup performance by compensating for incomplete retention on a single column and provided a more practical purification strategy for multi-vitamer analysis. Nevertheless, the final validation results confirmed that cumulative purification loss remained strongly vitamer-dependent, particularly for OHCbl, which continued to represent a major limitation of the current workflow.

### 3.4. Method Validation Results

The IDLs of the vitamers are presented in Table 2 and were 0.6 µg/L for OHCbl and 0.5 µg/L for CNCbl, AdoCbl, and MeCbl. Under the present experimental conditions, and taking into account sample preparation concentration factors and matrix effects as reflected by the spike-recovery results, the validated LODs were 0.5 μg/kg for CNCbl, 1.0 μg/kg for AdoCbl, and 0.75 μg/kg for MeCbl, with corresponding LOQs of 1.5 μg/kg, 3.0 μg/kg, and 2.5 μg/kg, respectively. In contrast, OHCbl showed exceptionally low recovery, and its effective injection concentration in typical food samples fell below the IDL, precluding reliable detection under the current method conditions.

**Figure 5:**
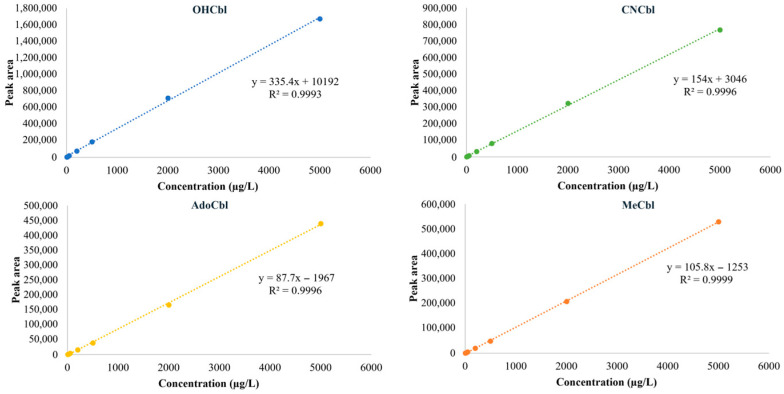
Calibration plots of OHCbl, CNCbl, AdoCbl, and MeCbl over the concentration range of 5–5000 µg/L. All analytes showed good instrumental linearity, with coefficients of determination (R^2^) higher than 0.999.

The calibration plots of the four vitamers are shown in Figure 5. All analytes exhibited good instrumental linearity over the tested concentration range of 5–5000 µg/L, consistent with the regression equations and R^2^ values summarized in Table 2. These results indicate that the instrumental response of the method was sufficiently linear for quantitative analysis within the tested range. However, good instrumental linearity alone did not guarantee equally reliable quantitative performance across all vitamers. In particular, although OHCbl showed acceptable linearity at the instrumental level, its extremely poor recovery during extraction and cleanup prevented reliable quantification in real samples under the current workflow. Therefore, linearity should be interpreted together with recovery and precision results when evaluating the practical applicability of the method.

Based on the spike-recovery results summarized in Table 2, CNCbl exhibited satisfactory recoveries of 84.5–91.4%, with CVs ranging from 2.4% to 8.8%, indicating good accuracy and precision under the current experimental conditions. This favorable performance can be attributed to the close physicochemical similarity between CNCbl and the internal standard ^13^C_7_-CNCbl, which likely contributed to better synchronization during extraction and cleanup. In contrast, AdoCbl and MeCbl showed lower recoveries of 58.5–63.2% and 67.4–71.1%, respectively, with corresponding CVs of 3.3–12.7% and 9.6–17.2%. These lower recoveries were mainly associated with greater losses during purification and incomplete compensation by the single internal standard. Their relatively higher variability was also consistent with the poorer light stability of these native vitamers, for which accidental light exposure during sample preparation may have contributed additional error. OHCbl showed extremely low recovery (<2.5%) and high variability (CV = 63.8%), most likely due to substantial losses on the purification columns and possible strong adsorption to sample components during extraction. As a result, accurate quantification of OHCbl in real samples could not be achieved under the current workflow, representing a major limitation of the present method. Taken together, the validation results support the practical applicability of the method for CNCbl, AdoCbl, and MeCbl within the present scope, while also showing that quantitative performance was clearly vitamer-dependent and that OHCbl could not be reliably quantified under the current workflow.

### 3.5. Quantitative Robustness in the Absence of Matched Isotopic Standards

While stable isotope dilution represents a rigorous strategy for correcting recovery loss and matrix effects, its routine implementation in multi-vitamer cobalamin analysis remains constrained by the limited availability and high cost of isotopically labeled standards [47]. Moreover, isotope dilution does not eliminate bias caused by degradation or interconversion that occurs before effective isotope equilibration. The following discussion is intended to interpret the quantitative implications of the validation results under the practical constraint that matched isotope-labeled standards were not available for all vitamers.

In the present study, the observed differences among vitamers are consistent with their distinct chemical stability, ionization behavior, and retention characteristics during cleanup. Because ^13^C_7_-CNCbl does not fully track the extraction, cleanup, and ionization behavior of AdoCbl and MeCbl, the present correction strategy should be regarded as partial procedural compensation rather than analyte-matched quantitative normalization. The validation results showed clearly vitamer-dependent performance, with satisfactory recovery for CNCbl, lower recoveries for AdoCbl and MeCbl, and extremely poor recovery for OHCbl. These findings indicate that quantitative robustness remained limited by analyte-specific behavior under the current workflow.

To improve quantitative consistency in routine analysis, a recovery-corrected quantification strategy based on concurrently analyzed QC materials was applied [64]. Although recovery-based correction is practically useful when true blank native matrices are difficult to obtain, it is less rigorous than full matrix-matched calibration or fully matched isotope-dilution analysis. Accordingly, the recovery-corrected quantification strategy used here should be regarded as a practical correction approach supported by the present validation results, rather than as a substitute for fully matched vitamer-specific isotope-dilution analysis.

### 3.6. Relationship to Conventional Cyanidation-Based Methods

This distinction is also important when comparing the present workflow with classical cyanidation-based methods. Conventional cyanidation procedures are primarily designed for the determination of total convertible vitamin B12 after chemical conversion of native cobalamins to cyanocobalamin, whereas the present method was specifically developed for cyanide-free, vitamer-resolved analysis aimed at preserving native vitamer information. Therefore, a direct one-to-one numerical comparison between the two approaches is not entirely straightforward.

At the same time, compared with conventional cyanidation-based methods used for total vitamin B12 determination, the present workflow avoids the use of potassium or sodium cyanide, thereby reducing reagent-handling burden and the practical constraints associated with hazardous reagent procurement, use, and disposal. In addition, the method does not require the 30 min water-bath treatment at 100 °C commonly applied during cyanidation-based conversion, which simplifies sample preparation and improves workflow efficiency. However, given the absence of matched isotopic standards for all vitamers and the poor recovery of OHCbl, the present method should not be interpreted as a direct replacement for cyanidation-based reference procedures.

### 3.7. Positioning of the Present Workflow Within the Current Analytical Landscape

To place the present workflow in the context of current food cobalamin analysis, a concise comparison with representative analytical approaches is summarized in Table 3. These approaches broadly include classical cyanidation-based LC methods, which are primarily designed for total convertible vitamin B12 determination, and stable isotope dilution (SIDA) LC–MS/MS methods, which currently represent the most robust strategy for native vitamer-resolved analysis when full sets of matched isotope-labeled standards are available. Compared with classical cyanidation-based LC methods, the present workflow avoids hazardous cyanide reagents and is more suitable for preserving native vitamer information during sample preparation. Compared with SIDA LC–MS/MS, however, its quantitative robustness remains more limited because correction relied on partial procedural compensation by a single internal standard together with recovery-based QC adjustment, rather than on vitamer-matched isotope dilution.

As shown in Table 3, the three approaches differ not only in analytical target but also in practical applicability. Classical cyanidation-based LC methods remain advantageous for standardized total-vitamin B12 determination, particularly in fortified products and regulatory contexts, but they do not preserve native vitamer distribution. SIDA LC–MS/MS provides the strongest control of recovery loss and ion suppression and is therefore the most rigorous option for native vitamer-resolved quantification, although its broader routine implementation is constrained by the cost, limited availability, and technical burden associated with matched isotope-labeled standards. Under these practical constraints, the present cyanide-free UHPLC–MS/MS workflow may be more appropriately viewed as a fit-for-purpose complementary approach for routine or comparative native-vitamer analysis of CNCbl, AdoCbl, and MeCbl, especially when full isotope-matched standards are not readily available.

Accordingly, the main value of the present workflow lies in its practical balance between structural preservation and routine applicability rather than in benchmark-level quantitative performance. The workflow supports cyanide-free analysis of major cobalamin vitamers with acceptable instrumental linearity and workable performance for CNCbl, AdoCbl, and MeCbl within the present validation scope. At the same time, its limitations should be recognized clearly: quantitative performance remained vitamer-dependent, correction by ^13^C_7_-CNCbl was only partial, practical quantification further relied on recovery-based QC correction, and OHCbl could not be reliably quantified under the current workflow. In addition, the workflow was not directly benchmarked against an established vitamer-resolved reference method in the present study. Therefore, it should not be interpreted as demonstrating analytical equivalence or superiority to established SIDA LC–MS/MS approaches, but rather as a practical cyanide-free workflow for exploratory, comparative, or routine native-vitamer analysis under real laboratory constraints.

### 3.8. Determination of Practical Samples

Based on the detection method established in this study, we analyzed the contents of CNCbl, AdoCbl, and MeCbl in 43 representative samples, including 24 plant-derived samples and 19 animal-derived samples. The analyzed food matrices were intentionally selected to represent different practical scenarios for method applicability assessment. Plant-derived foods were included as matrices expected to contain no detectable target cobalamins, animal livers as matrices with biologically plausible high cobalamin levels, and marine samples as matrices known to show relatively high but variable cobalamin contents. This sample set was therefore used to examine whether the present workflow could provide practically interpretable results across negative, high-level, and compositionally variable food matrices within the current validation scope. As shown in Table 4, no vitamers were detected in any of the plant-derived samples, including wheat, corn, millet, peanuts, and wild jujube tea. This finding is consistent with previous reports indicating the absence of detectable cobalamin in most plant-based foods [2,41].

The quantitative results revealed that animal livers contain high levels of cobalamins, with the total content of three vitamers (excluding OHCbl) in chicken liver, pork liver, and lamb liver reaching 16.32 μg/100 g, 30.45 μg/100 g, and 64.67 μg/100 g, respectively. These values are consistent with previous literature reports [20,23] and further confirm that animal liver is the primary storage site of vitamin B12 [4]. Notably, AdoCbl accounted for 55–85% of the total cobalamins, aligning with findings from Czerwonka et al. [22] and indicating that AdoCbl was the predominant detected vitamer in liver in the present dataset. Furthermore, oysters contained a total cobalamin concentration of 48.53 μg/100 g, which is substantially lower than the 421.2 mg/kg reported previously [26]. Given the very large discrepancy between μg/100 g and mg/kg units, a possible unit inconsistency in the earlier report cannot be excluded. If interpreted on a comparable unit basis, the values fall within a similar magnitude range, suggesting that part of the apparent discrepancy may arise from reporting conventions rather than true biological divergence. Sea cucumbers also exhibited detectable levels of multiple vitamers; however, pronounced variability was observed among samples. Such dispersion may reflect intrinsic biological heterogeneity related to species differences, feeding ecology, and environmental conditions in wild marine organisms. Additionally, when concentrations approach the limit of quantification (LOQ), minor analytical fluctuations can amplify relative variability, further contributing to poor reproducibility. These findings are consistent with the generally high inter-study variation reported for aquatic products and underscore the importance of structural confirmation and methodological control in marine cobalamin analysis.

**Figure 6:**
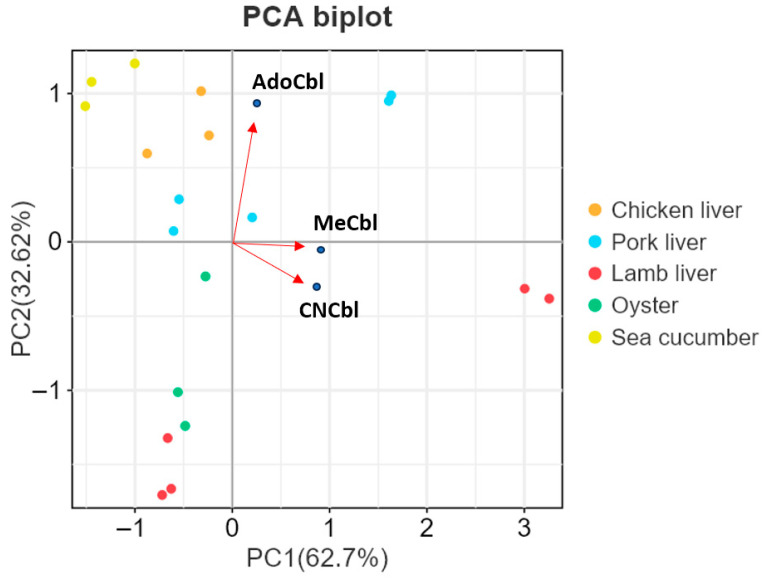
PCA biplot of the 19 animal-derived samples based on the contents of CNCbl, AdoCbl, and MeCbl. Points represent individual samples, and arrows indicate the loading directions of the three cobalamin variants. PC1 and PC2 explained 62.7% and 32.62% of the total variance, respectively.

To further visualize differences in cobalamin profiles among animal-derived food matrices, PCA was performed based on the contents of CNCbl, AdoCbl, and MeCbl in the 19 animal-derived samples (Figure 6). PC1 and PC2 explained 62.7% and 32.62% of the total variance, respectively. The PCA biplot showed a matrix-dependent distribution pattern among the samples. Chicken liver, pork liver, lamb liver, oyster, and sea cucumber samples occupied different regions in the PCA space, indicating differences in their cobalamin compositions. According to the loading vectors, PC2 was mainly associated with AdoCbl, whereas PC1 was mainly influenced by MeCbl and CNCbl. Sea cucumber samples occupied a distinct region in the PCA space, indicating a compositional pattern different from the other matrices. Lamb liver samples were clearly separated from the other matrices along the positive direction of PC1, which may be related to their relatively high levels of MeCbl and CNCbl. These results further demonstrated the applicability of the established method for differentiating cobalamin profiles among animal-derived foods.

The practical sample analysis provided matrix-level observations that were broadly compatible with the current validation results and with previously reported concentration ranges and vitamer-distribution trends in the literature. However, because no direct comparison with an established vitamer-resolved analytical method was performed in the present study, these results should be interpreted only as indirect support for practical plausibility rather than as evidence of analytical equivalence or validated comparative performance. Future work should focus on improving OHCbl recovery, further optimizing cleanup selectivity, and incorporating matched isotopic standards and direct comparative benchmarking to strengthen quantitative trueness and cross-method comparability.

## 4. Conclusions

A cyanide-free UHPLC-MS/MS workflow was developed for the analysis of major cobalamin vitamers in foods under light-controlled conditions. The method enabled instrumental separation and detection of CNCbl, AdoCbl, MeCbl, and OHCbl, and the validation results supported practical applicability for CNCbl, AdoCbl, and MeCbl within the present scope of the study. However, method performance was clearly vitamer-dependent, and OHCbl showed very poor recovery and could not be reliably quantified in real samples under the current workflow.

Compared with conventional cyanidation-based methods for total cobalamin determination, the present approach avoids the use of cyanide reagents and simplifies sample preparation by omitting the high-temperature conversion step. At the same time, its analytical target is native vitamer-resolved analysis rather than cyanide-converted total cobalamin. Therefore, the method should be regarded as a practical cyanide-free workflow for routine and comparative analysis of major cobalamin vitamers, rather than as a direct replacement for cyanidation-based reference procedures or fully matched isotope-dilution assays.

Application to food samples showed no detectable target cobalamins in the tested plant-derived foods and distinct vitamer profiles in animal-derived foods, with AdoCbl predominating in liver. Overall, this study describes a cyanide-free workflow that may be useful for practical native-vitamer analysis under the current validation scope. However, because the workflow was not benchmarked against an established vitamer-resolved analytical method, it should not be interpreted as demonstrating reference-level accuracy, validated superiority, or direct analytical equivalence to existing benchmark approaches. Further optimization and direct comparative validation remain necessary, particularly for OHCbl and for broader quantitative harmonization across vitamers.

## Figures and Tables

**Table 1:** UHPLC-MS/MS parameters of the proposed method to determine cobalamins.

Items			Parameters			
UHPLC	Apparatus		Agilent 1290 Infinity II			
	Injection volume		2 μL			
	Column		AQ C18 2.7 µm (2.1 × 100 mm)			
	Oven temperature		40 °C			
	Flow		0.2 mL/min			
	Mobile phase A		5 mM ammonium formate aqueous solution			
	Mobile phase B		0.1% formic acid/acetonitrile			
	Eluent gradient		Time (min)	A (%)		B (%)
			0	90		10
			0.5	90		10
			2	80		20
			6	10		90
			6.5	10		90
			7	90		10
			8	90		10
MS/MS	Apparatus		Agilent 6475			
	Ion source		AJS ESI+			
	Dryer temperature		280 °C			
	Dryer flow		13 L/min			
	Atomizing gas pressure		35 psi			
	Sheath temperature		320 °C			
	Sheath gas flow		10 L/min			
	Capillary voltage		4000 V			
	Nozzle voltage		1500 V			
	Scanning mode		Multi-Reaction Monitoring			
	Compound	Characteristic ion pair (*m*/*z*)		DP (V)	CE (eV)	Dwell time (ms)
	* ^13^C_7_-CNCbl	Quantitative	682.1 → 153.9	140	45	100
		Qualitative	682.1 → 365.8	140	25	100
	CNCbl	Quantitative	678.7 → 147.1	160	45	50
		Qualitative	678.7 → 359.2	160	25	50
	AdoCbl	Quantitative	790.8 → 147.1	140	45	50
		Qualitative	790.8 → 359.2	120	35	50
	MeCbl	Quantitative	673.3 → 147.1	140	25	50
		Qualitative	673.3 → 359.2	140	30	50
	OHCbl	Quantitative	664.9 → 147.1	140	30	50
		Qualitative	664.9 → 359.2	140	15	50

* The internal standard; DP: declustering potential; CE: collision energy.

**Table 2:** Sensitivity, linearity, spike recovery tests of the vitamers in soybean protein powder (*n* = 3).

Compound	IDL (µg/L)	LOD (µg/kg)	LOQ (µg/kg)	Linearity Range (µg/L)	Standard Curve Regression Equation	R^2^	Spike Recovery					
							2 μg/100 g		10 μg/100 g		50 μg/100 g	
							Average (%)	CV (%)	Average (%)	CV (%)	Average (%)	CV (%)
OHCbl	0.6	NA	NA	5–5000	y = 335.4x + 10,192	0.9993	NA	NA	2.5	23.2	2.3	63.8
CNCbl	0.5	0.5	1.5	5–5000	y = 154x + 3046	0.9996	90.6	6.5	91.4	2.4	84.5	8.8
AdoCbl	0.5	1	3	5–5000	y = 87.7x − 1967	0.9996	63.2	3.3	58.5	3.6	60.9	12.7
MeCbl	0.5	0.75	2.5	5–5000	y = 105.8x − 1253	0.9999	71.1	9.6	67.4	17.2	70.8	15.1

IDL: instrumental detection limit; LOD: Limit of Detection; LOQ: Limit of Quantitation; CV: coefficient of variation; NA: not available.

**Table 3:** Structured comparison of the present workflow with representative analytical approaches for food cobalamin analysis.

Comparison Item	AOAC/Classical Cyanidation-Based LC [37,38,62]	Stable Isotope Dilution LC–MS/MS [42]	Present Cyanide-Free UHPLC–MS/MS Workflow
Primary analytical target	Total convertible vitamin B12 after conversion to CNCbl	Native vitamer-resolved cobalamin analysis	Native-vitamer-oriented analysis under the current validation scope
Cyanidation required	Yes	No	No
Simultaneous native vitamer analysis	No	Yes	Yes, mainly for CNCbl, AdoCbl, and MeCbl within the current scope
Matrix-effect / recovery correction strategy	Usually external calibration or non-isotopic calibration; limited correction for matrix effects	Vitamer-matched isotope-labeled internal standards; strongest correction for recovery loss and ion suppression	Partial procedural compensation by a single internal standard, combined with recovery-based QC correction
Representative quantitative robustness	Good for standardized total-B12 determination in fortified products	Highest among the compared approaches	Moderate; fit-for-purpose under practical constraints
Representative validation performance	Good regulatory robustness for total B12	High trueness and strong quantitative robustness	Good linearity; satisfactory recovery for CNCbl, moderate for AdoCbl and MeCbl; OHCbl not reliably quantified
Scope of applicability	Routine total-B12 analysis, especially fortified products	Benchmark native vitamer analysis when full matched isotopic standards are available	Routine or comparative native-vitamer analysis when full matched isotope-labeled standards are not readily available
Main strengths	Standardized; suitable for regulatory total-B12 determination	Best structural confirmation and quantitative correction	Cyanide-free; preserves native vitamer information better; practically applicable under routine laboratory constraints
Main limitations	Destroys native vitamer distribution; requires hazardous cyanide reagents	High cost, limited availability, and technical burden of matched labeled standards	Quantitative robustness remains vitamer-dependent; single internal standard provides only partial procedural compensation; OHCbl performance poor

**Table 4:** The contents of vitamers in representative food samples (µg/100 g, mean ± SD).

Food Matrix	CNCbl	AdoCbl	MeCbl
Wheat flour (*n* = 8)	ND	ND	ND
Maize flour (*n* = 3)	ND	ND	ND
Millet (*n* = 3)	ND	ND	ND
Peanut (*n* = 3)	ND	ND	ND
Wild jujube tea (*n* = 7)	ND	ND	ND
Chicken liver (*n* = 3)	2.12 ± 1.10	9.28 ± 1.42	4.92 ± 1.44
Pork liver (*n* = 5)	3.15 ± 1.10	20.30 ± 4.46	7.01 ± 3.96
Lamb liver (*n* = 5)	2.97 ± 2.70	54.27 ± 3.95	7.43 ± 5.34
Oyster (*n* = 3)	1.76 ± 0.75	42.91 ± 8.66	3.86 ± 0.47
Sea cucumber (*n* = 3)	1.17 ± 0.60	1.58 ± 0.95	1.93 ± 1.22

## Data Availability

The original contributions presented in this study are included in the article. Further inquiries can be directed to the corresponding author.

## Abbreviations

The following abbreviations are used in this manuscript:

AdoCbl	5′-Deoxyadenosylcobalamin
B12	Vitamin B12
CE	Collision Energy
CNCbl	Cyanocobalamin
CV	Coefficient of Variation
DP	Declustering Potential
HLB	Hydrophilic–Lipophilic Balance
IAC	Immunoaffinity Column
IDL	Instrument Detection Limit
LOD	Limit of Detection
LOQ	Limit of Quantification
MeCbl	Methylcobalamin
OHCbl	Hydroxocobalamin
QC	Quality Control
SIC	Selected Ion Chromatogram
SIDA	Stable Isotope Dilution Assay
SPE	Solid-Phase Extraction
TIC	Total Ion Chromatogram
UHPLC	Ultra-High-Performance Liquid Chromatography
UHPLC–MS/MS	Ultra-High-Performance Liquid Chromatography–Tandem Mass Spectrometry
